# Supplementary material for: Pimasertib Versus Dacarbazine in Patients With Unresectable NRAS-Mutated Cutaneous Melanoma: Phase II, Randomized, Controlled Trial with Crossover
Source: Cancers (Basel). 2020 Jun 29;12(7):1727. doi: 10.3390/cancers12071727 (PMC7408351; doi:10.3390/cancers12071727)
Supplement: Supplementary file 1 [file cancers-12-01727-s001.pdf]

Supplementary Materials: Pimasertib Versus Dacarbazine in Patients with Unresectable *NRAS*-Mutated Cutaneous Melanoma: Phase II, Randomized, Controlled Trial with Crossover

Celeste Lebbé, Caroline Dutriaux, Thierry Lesimple, Willem Kruit, Joseph Kerger, Luc Thomas, Bernard Guillot, Filippo de Braud, Claus Garbe, Jean-Jacques Grob, Carmen Loquai, Virginia Ferraresi, Caroline Robert, Paul Vasey, Robert Conry, Richard Isaacs, Enrique Espinosa, Armin Schueler, Giorgio Massimini and Brigitte Dréno

Information S1. Inclusion criteria

For inclusion in the trial, all of the following inclusion criteria were to be fulfilled:

1. Patients with measurable, histologically or cytologically confirmed, unresectable locally advanced or metastatic cutaneous melanoma (stage IIIc or IV [M1a-c]) *NRAS* mutated. If *NRAS* mutational status was unknown at screening, it had to be prospectively defined before inclusion. If *NRAS* mutational status was already known before screening, it was retrospectively confirmed after inclusion by the Sponsor.
2. Tumour lesions amenable to biopsy or available tumour tissue as archival samples.
3. Age ≥18 years.
4. Had read and understood the informed consent form and was willing and able to give informed consent. Fully understood requirements of the trial and was willing to comply with all trial visits and assessments.
5. Women of childbearing potential had to have a negative blood pregnancy test at the Screening Visit. For the purposes of this trial, women of childbearing potential were defined as: "All female patients after puberty unless they are postmenopausal for at least 2 years or are surgically sterile".
6. Female patients of childbearing potential and male patients with female partners of childbearing potential had to be willing to avoid pregnancy by using an adequate method of contraception for 2 weeks prior to, during, and 4 weeks after the last dose of trial treatment. Effective contraception was defined as the method of contraception with a failure rate of less than 1% per year. Adequate contraception for female patients or female partners of male patients was defined as follows: two barrier methods or one barrier method in combination with an intrauterine device or oral contraception (Global CTPA 3 and Local [Germany] CTPA 1).

Information S2. Exclusion criteria

Patients were not eligible for this trial if they fulfilled any of the following exclusion criteria:

1. Had previous systemic treatment for locally advanced or metastatic cutaneous melanoma (excluding adjuvant treatment).
2. Had non-measurable lesions or disease not evaluable by Response Evaluation Criteria in Solid Tumors (RECIST) version 1.1.
3. Had an Eastern Cooperative Oncology Group performance status (ECOG PS) >1.
4. Had bone marrow impairment as evidenced by haemoglobin <10.0 g/dL, neutrophil count <1.5 × 10^9/L, platelets <100 × 10^9/L.
5. Had renal impairment as evidenced by calculated creatinine clearance <60 mL/min (according to the Cockcroft-Gault formula).

6. Had liver function abnormality as defined by total bilirubin >1.5 × upper limit of normal (ULN), or aspartate aminotransferase (AST)/alanine aminotransferase (ALT) >2.5 × ULN; for patients with liver involvement, AST/ALT >5 × ULN.
7. Had significant cardiac conduction abnormalities, including corrected QT interval (QTc) prolongation of >480 ms and/or pacemaker or clinically relevant impaired cardiovascular function (New York Heart Association class III/IV).
8. Had hypertension uncontrolled by medication.
9. Had retinal degenerative disease (hereditary retinal degeneration or age-related macular degeneration), history of uveitis, or history of retinal vein occlusion (RVO) or any eye condition that would be considered a risk factor for RVO (e.g. uncontrolled glaucoma or ocular hypertension).
10. Had known active central nervous system metastases unless previously radiotherapy treated, stable by computed tomography scan for at least 3 months without evidence of cerebral oedema and no requirements for corticosteroids or anticonvulsants.
11. History of difficulty in swallowing, malabsorption or other chronic gastrointestinal disease, or conditions that could hamper compliance and/or absorption of the tested product.
12. Known human immunodeficiency virus positivity, active hepatitis C or active hepatitis B.
13. Had undergone surgical intervention within 28 days from Day 1 of trial treatment.
14. Had received extensive prior radiotherapy on more than 30% of bone marrow reserves, or prior bone marrow/stem-cell transplantation within 5 years from Day 1 of trial treatment.
15. Had history of any other significant medical disease such as major gastric or small bowel surgery, recent drainage of significant volumes of ascites or pleural effusion, or had a psychiatric condition that could have impaired the patients' well-being or precluded full participation in the trial.
16. Had known hypersensitivity to dacarbazine.
17. Was a pregnant or nursing female.
18. Participated in another clinical trial within the previous 28 days.
19. Had creatine phosphokinase (CPK) level at Baseline National Cancer Institute Common Terminology Criteria for Adverse Events (NCI-CTCAE) grade ≥2 (i.e. >2.5 × ULN), and/or had a previous history of myositis or rhabdomyolysis (Global CTPA 3 and Local [Germany] CTPA 1).
20. Was suitable for trial treatment with an approved BRAF inhibitor (Local [Germany] CTPA 1) or anti-human CTLA-4 (CD152) monoclonal antibodies (such as ipilimumab) (Global CTPA 4).

Information S3. De-escalation or discontinuation criteria

Criteria for de-escalation or discontinuation of pimasertib were based on the occurrence of: serious retinal detachment with change in visual acuity (≥15 points the on Early Treatment Diabetic Retinopathy Study score) or RVO; and grade 3/4 skin disorders, diarrhea, QT/QTc prolongation or haematological toxicity. If symptoms resolved completely/partially after dose interruption and appropriate symptomatic treatment, pimasertib could be reinitiated at 45 mg twice daily (bid; reduction to 30 mg bid was permitted in some cases). If there was no improvement after 2 weeks, pimasertib was permanently discontinued. Patients who developed grade ≥3 CPK elevation had to interrupt pimasertib treatment and continue weekly CPK monitoring. Treatment could be resumed at a lower dose if the level decreased to ≤2.5 × ULN within 2 weeks. Pimasertib was permanently discontinued if ≥3 dose interruptions were required.

Table S1. *NRAS* mutations identified in enrolled patients (ITT analysis set).

**Mutations**	**DTIC (n = 64)**		**Pimasertib (n = 130)**	
***NRAS* Mutations, *n* (%) ***	**Central (n = 60)**	**Local (n = 42)**	**Central (n = 125)**	**Local (n = 89)**
G12D	0	0	1 (1)	2 (2)
Q61H	3 (5)	2 (5)	7 (6)	2 (2)
Q61K	20 (33)	18 (43)	49 (39)	28 (32)
Q61L	4 (7)	2 (5)	19 (15)	14 (16)
Q61R	30 (50)	17 (40)	47 (38)	39 (44)
Other	6 (10)	3 (7)	10 (8)	4 (5)

Abbreviations: DTIC, dacarbazine; ITT, intent-to-treat. * All data have been rounded up to the nearest whole number.

Table S2. PFS–primary and sensitivity analyses (ITT analysis set) *.

Analysis	Primary Analysis				Sensitivity Analysis †			
	Investigator Assessment		Independent Central Read ‡		Investigator Assessment		Independent Central Read	
	DTIC (n = 64)	Pimasertib (n = 130)	DTIC (n = 64)	Pimasertib (n = 130)	DTIC (n = 64)	Pimasertib (n = 130)	DTIC (n = 64)	Pimasertib (n = 130)
Events, n (%)	58 (91)	86 (66)	50 (78)	82 (63)	59 (92)	105 (81)	32 (50)	60 (46)
Censored, n (%)	6 (9)	44 (34)	14 (22)	48 (37)	5 (8)	25 (19)	32 (50)	70 (54)
Median PFS (95% CI), weeks	7 (6–12)	13 (12–18)	6 (6–12)	13 (12–18)	7 (6–12)	17 (12–19)	13 (7–19)	18 (12–19)
p-value	p = 0.0022		p = 0.0195		p = 0.0004		p = 0.1454	
HR (95% CI)	0.59 (0.42–0.83)		0.65 (0.45–0.94)		0.56 (0.40–0.77)		0.72 (0.46–1.12)	

Abbreviations: CI, confidence interval; DTIC, dacarbazine; HR, hazard ratio; ITT, intent-to-treat; PD, progressive disease; PFS, progression-free survival. * All data have been rounded up to the nearest whole number, except for HRs and P-values. † Sensitivity analysis including all deaths and all scans considers all imaging data that was collected from patients who discontinued trial treatment without PD ‡ Sensitivity analysis.

Table S3. Summary of TEAEs (safety analysis set).

Patients, *n* (%) *	DTIC (*n* = 61) †	Pimasertib (*n* = 130)	Pimasertib Crossover (*n* = 41)
Any TEAE	60 (98)	130 (100)	41 (100)
Any grade ≥3 TEAE	25 (41)	111 (85)	36 (88)
Any drug-related TEAE	54 (89)	130 (100)	41 (100)
Any drug-related grade ≥3 TEAE	17 (28)	100 (77)	35 (85)
Any serious TEAE	12 (20)	74 (57)	26 (63)
Any drug-related serious TEAE	4 (7)	59 (45)	20 (49)
Any fatal drug-related serious TEAE	1 (2)	2 (2)	2 (5)
Any TEAE leading to dose reduction, interruption or delay	16 (26)	105 (81)	32 (78)
Any TEAE leading to permanent treatment discontinuation	3 (5)	61 (47)	16 (39)
Any drug-related TEAE leading to permanent treatment discontinuation	0	56 (43)	13 (32)

Abbreviations: DTIC, dacarbazine; TEAE, treatment-emergent adverse event. * All data have been rounded up to the nearest whole number. † Includes events reported on DTIC treatment until 33 days after the last dose of DTIC. Any events that started after patient had crossed over to pimasertib treatment (*n* = 41) are counted in the pimasertib crossover group.

Table S4. TEAEs occurring in >25% of patients in any treatment group (safety analysis set).

Patients, *n* (%) *	DTIC (*n* = 61)	Pimasertib (*n* = 130)	Pimasertib crossover † (*n* = 41)
Any TEAE (≥1 event)	60 (98)	130 (100)	41 (100)
Diarrhoea	10 (16)	107 (82)	31 (76)
Blood CPK increased	3 (5)	89 (68)	29 (71)
Oedema peripheral	6 (10)	60 (46)	18 (44)
Retinal detachment	0	58 (45)	16 (39)
Dermatitis acneiform	0	48 (37)	9 (22)
Nausea	25 (41)	47 (36)	17 (41)
Rash	5 (8)	46 (35)	18 (44)
Fatigue	23 (38)	40 (31)	11 (27)
Asthenia	13 (21)	39 (30)	10 (24)
Vomiting	14 (23)	31 (24)	17 (42)
Constipation	21 (34)	25 (19)	7 (17)

Abbreviations: AE, adverse event; CPK, creatinine phosphokinase; DTIC, dacarbazine; TEAE, treatment-emergent adverse event. * All data have been rounded up to the nearest whole number. † Patients receiving pimasertib crossover had previously progressed following treatment with DTIC and crossed over to pimasertib. AEs were reported under pimasertib crossover if the AE started on or after the first dose of pimasertib. The event was also reported under DTIC if the AE was recorded within 33 days of starting pimasertib.

Table S5. TEAEs of special interest (safety analysis set)*.

**Ocular AESIs**‡	**DTIC (*n* = 61)**	**Pimasertib (*n* = 130)**	**Pimasertib Crossover** † **(*n* = 41)**
Number of patients with at least one ocular AESI, *n* (%)	19 (31)	76 (58)	21 (51)
Number of patients with at least one SRD ocular AESI, *n* (%)	19 (31)	76 (59)	21 (51)
Number of patients with at least one RVO ocular AESI, *n* (%)	0	5 (4)	2 (5)
Number of patients with at least one ocular AESI leading to treatment delay/interruption of >3 days, *n* (%)	0	26 (20)	7 (17)
Number of patients with at least one SRD ocular AESI leading to treatment discontinuation, *n* (%)	0	12 (9)	3 (7)
Number of patients with reversible ocular AESI, *n* (%)	18 (95)	70 (92)	20 (95)

**CPK-Related AESIs**	**DTIC (*n* = 61)**	**Pimasertib (*n* = 130)**	**Pimasertib Crossover** † **(*n* = 41)**
Number of patients with at least one (any grade) CPK increase event, *n* (%)	17 (28)	74 (57)	24 (59)
Time to first (any grade) CPK increase event, median (range), weeks	14 (7–37)	3 (1–18)	3 (1–15)
Duration of first (any grade) CPK increase event, median (range), weeks	1 (1–5)	1 (0.3–20)	1 (1–9)
Number of patients with at ≥1 CPK increase event leading to treatment delay/interruption of >3 days, *n* (%)	0	40 (31)	13 (32)
Number of patients with at least one CPK increase event leading to treatment discontinuation, *n* (%)	0	17 (13)	1 (2)
Number of patients with reversible CPK increase event, *n* (%)	15 (88)	70 (95)	22 (92)

Abbreviations: AE, adverse event; AESI, adverse event of special interest; CPK, creatinine phosphokinase; DTIC, dacarbazine; RVO, retinal vein occlusion; SRD, serous retinal detachment; TEAE, treatment-emergent adverse event. * All data have been rounded up to the nearest whole number. † Patients receiving pimasertib crossover had previously progressed following treatment with DTIC and crossed over to pimasertib. AEs were reported under pimasertib crossover if the AE started on or after the first dose of pimasertib. The event was also reported under DTIC if the AE was recorded within 33 days of starting pimasertib crossover treatment. ‡ Ocular AESIs were limited to SRD and RVO.Rate of patients with an ocular event = number of patients with ocular event of interest/exposure (patient years).

Table S6. Quality-of-life measures in patients at baseline and end of treatment (ITT analysis set) *.

Measures	DTIC (*n* = 64)	Pimasertib (*n* = 130)
**PWB**		
Baseline, mean (SD)	24 (5)	23 (5)
End of treatment, mean (SD)	20 (5)	20 (6)
**SWB**		
Baseline, mean (SD)	21 (6)	23 (5)
End of treatment, mean (SD)	21 (5)	22 (5)
**EWB**		
Baseline, mean (SD)	15 (5)	16 (5)
End of treatment, mean (SD)	15 (6)	17 (5)
**FWB**		
Baseline, mean (SD)	16 (6)	18 (6)
End of treatment, mean (SD)	14 (5)	16 (7)
**MS**		
Baseline, mean (SD)	53 (9)	53 (8)
End of treatment, mean (SD)	49 (9)	50 (9)
**MSS**		
Baseline, mean (SD)	26 (5)	26 (7)
End of treatment, mean (SD)	26 (6)	26 (7)
**FACT-M TS**		
Baseline, mean (SD)	126 (24)	132 (22)
End of treatment, mean (SD)	119 (22)	123 (26)
**FACT-M TOI**		
Baseline, mean (SD)	90 (19)	94 (18)
End of treatment, mean (SD)	84 (17)	85 (21)
**FACT-G TS**		
Baseline, mean (SD)	75 (15)	80 (16)
End of treatment, mean (SD)	70 (15)	74 (17)

Abbreviations: DTIC, dacarbazine; EWB, Emotional Well-Being; FACT-G TS, Functional Assessment of Cancer Therapy–General Total Score; FACT-M TOI, Functional Assessment of Cancer Therapy–Melanoma Trial Outcome Index; FACT-M TS, Functional Assessment of Cancer Therapy–Melanoma Total Score; FWB, Functional Well-Being; ITT, intent-to-treat; MS, Melanoma Subscale; MSS, Melanoma Surgery Scale; PWB, Physical Well-Being; SD, standard deviation; SWB, Social/Family Well-Being. * All data have been rounded up to the nearest whole number. The EWB, FWB, MS, MSS, PWB and SWB are single-item scales. FACT-M TS = PWB + SWB + EWB + FWB + MS. FACT-M TOI = PWB + FWB + MS. FACT-G TS = PWB + SWB + EWB + FWB.

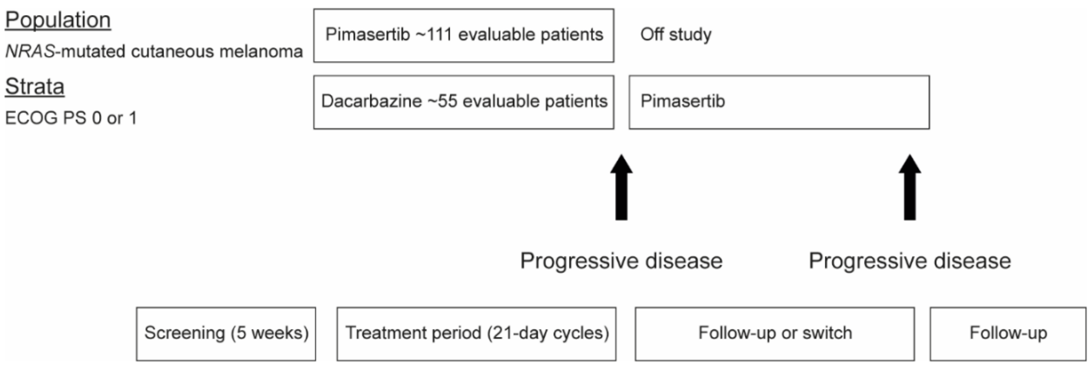

Figure S1. Trial design. Abbreviations: DTIC, dacarbazine; ECOG PS, Eastern Cooperative Oncology Group performance status.

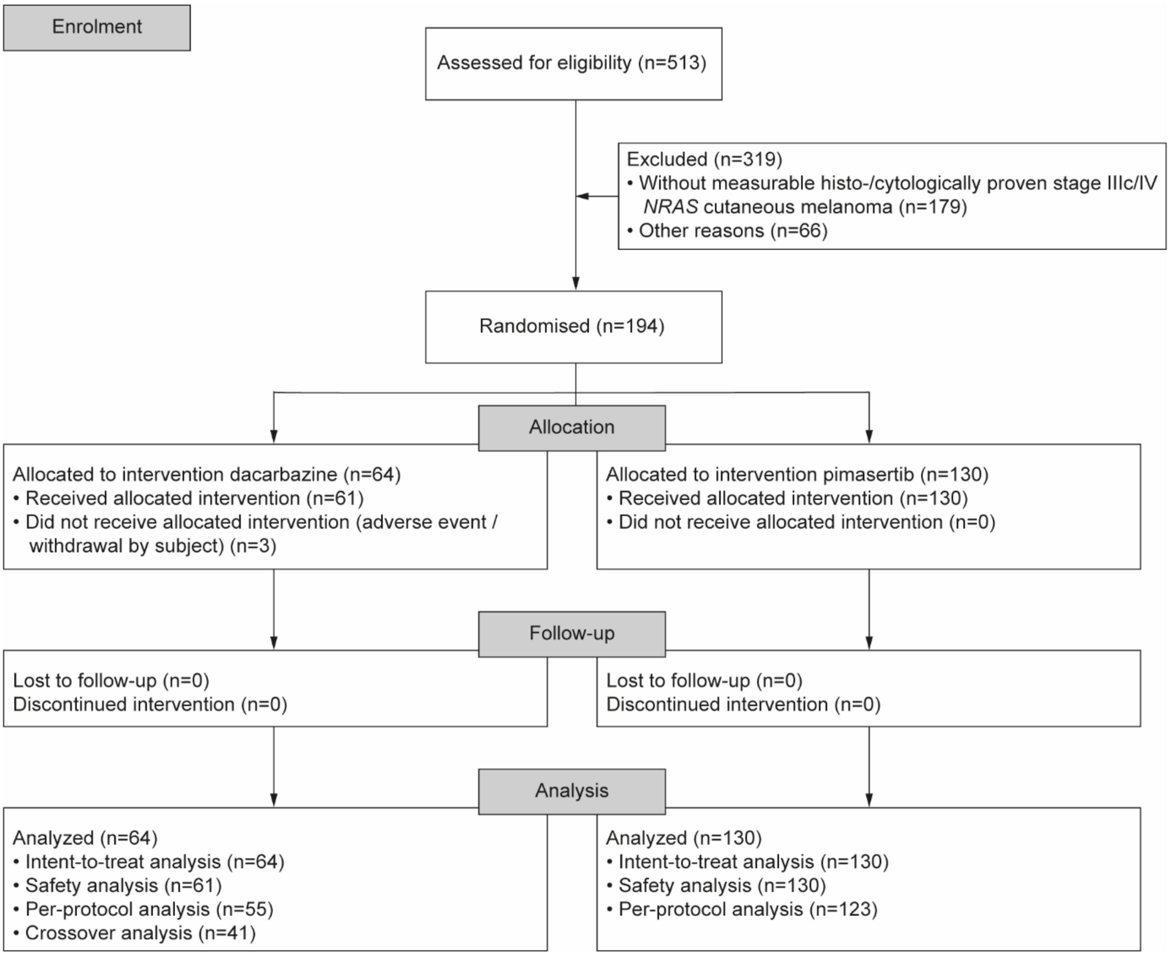

Figure S2. Disposition of patients through the trial (CONSORT diagram). Abbreviations: CONSORT, Consolidated Standards of Reporting Trials; DTIC, dacarbazine.

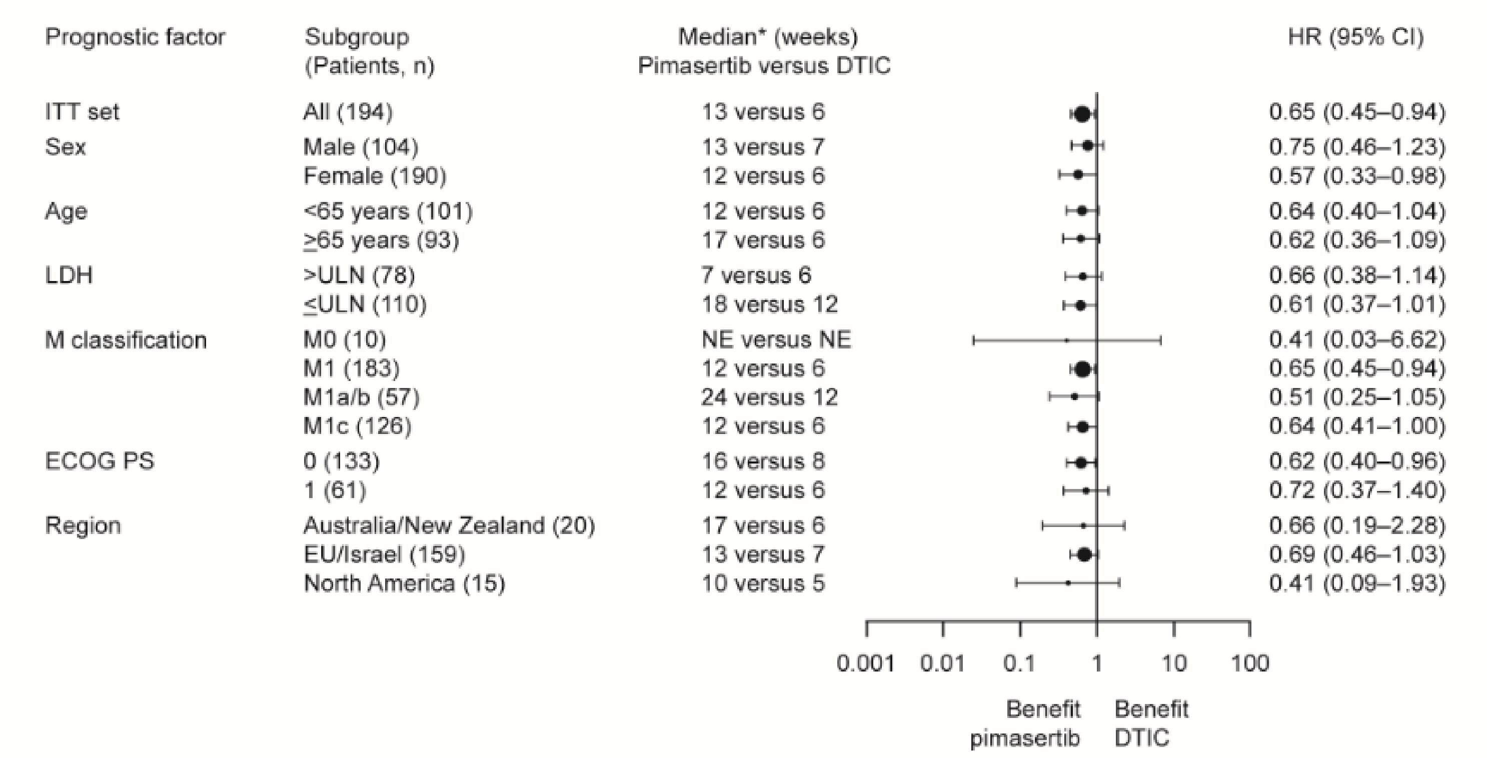

Figure S3. Forest plots for PFS: pimasertib versus DTIC (centrally assessed endpoints; ITT analysis set). Abbreviations: CI, confidence interval; DTIC, dacarbazine; ECOG PS, European Cooperative Oncology Group performance status; EU, European Union; HR, hazard ratio; ITT, intent-to-treat; LDH, lactate dehydrogenase; NE, not evaluable; PFS, progression-free survival; PD, progressive disease; ULN, upper limit of normal.
* Median numbers rounded to nearest whole number.

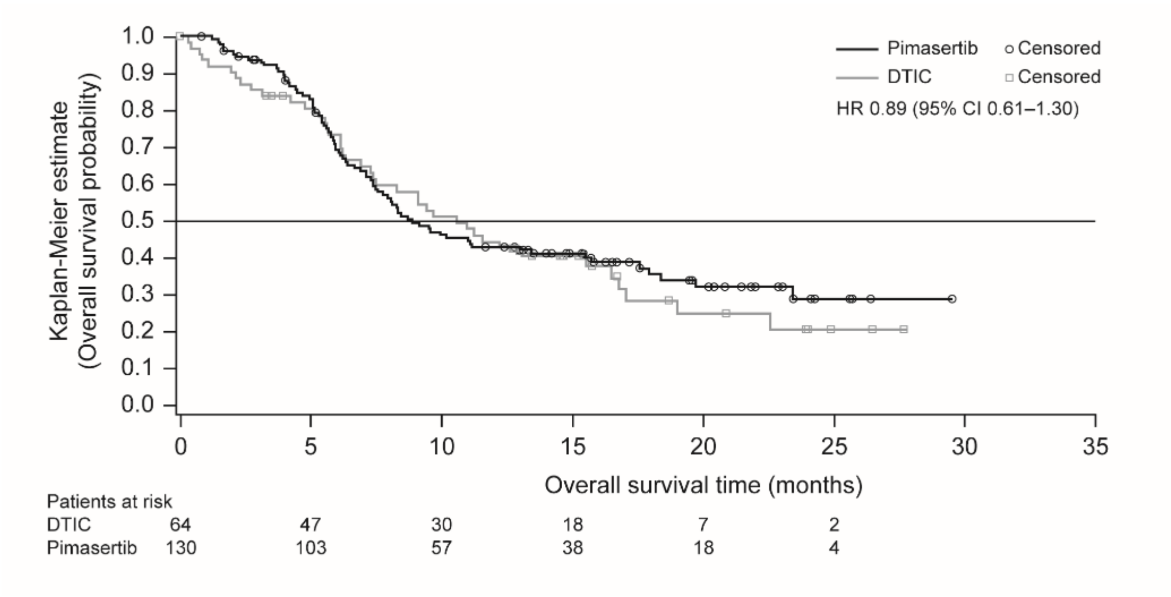

Figure S4. Kaplan-Meier plot of overall survival: pimasertib versus DTIC, based on investigator reading of data (ITT analysis set). Abbreviations: CI, confidence interval; DTIC, dacarbazine; HR, hazard ratio; ITT, intent-to-treat.

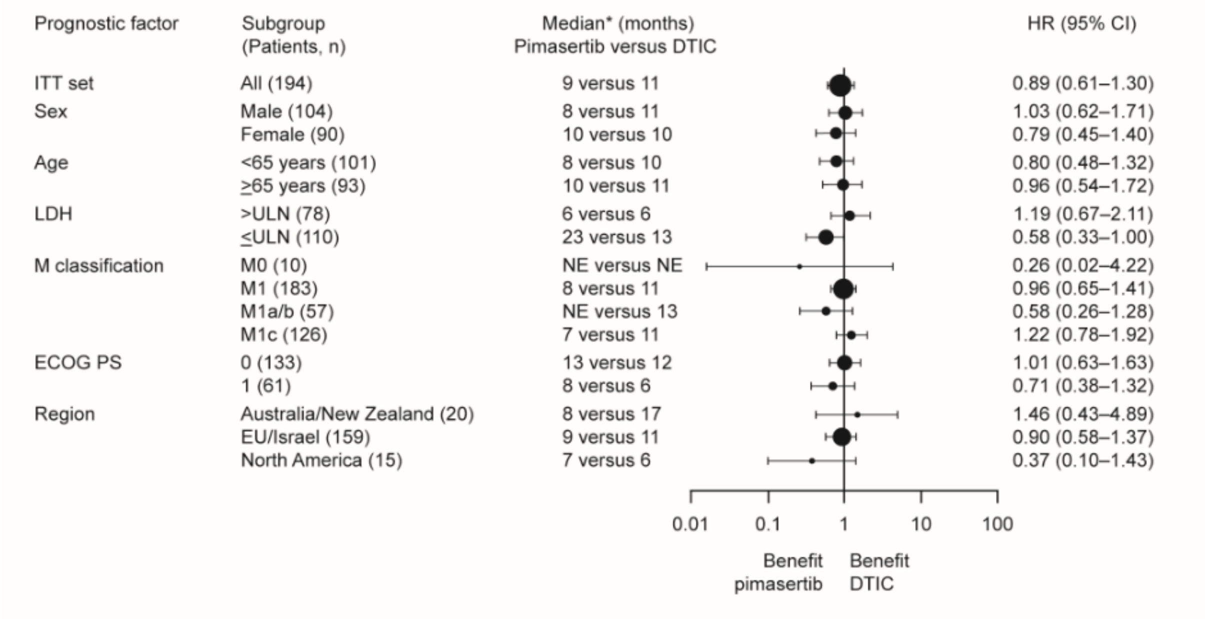

Figure S5. Forest plot of effect of prognostic factors on overall survival (ITT analysis set). Abbreviations: CI, confidence interval; DTIC, dacarbazine; ECOG PS, Eastern Cooperative Oncology Group performance status; EU, European Union; HR, hazard ratio; ITT, intent-to-treat; LDH, lactate dehydrogenase; NE, not evaluable; ULN, upper limit of normal. * Median numbers rounded to nearest whole number.
